# Brain Imaging Biomarkers for Chronic Pain

**DOI:** 10.3389/fneur.2021.734821

**Published:** 2022-01-03

**Authors:** Zhengwu Zhang, Jennifer S. Gewandter, Paul Geha

**Affiliations:** ^1^Department of Statistics and Operations Research, University of North Carolina at Chapel Hill, Chapel Hill, NC, United States; ^2^Anesthesiology and Perioperative Medicine, School of Medicine and Dentistry, University of Rochester, Rochester, NY, United States; ^3^Department of Psychiatry, School of Medicine and Dentistry, University of Rochester, Rochester, NY, United States; ^4^Department of Neurology, School of Medicine and Dentistry, University of Rochester, Rochester, NY, United States; ^5^Del Monte Neuroscience Institute, University of Rochester, Rochester, NY, United States

**Keywords:** chronic pain, neuroimaging, limbic brain, diagnosis, prognosis, biomarkers

## Abstract

The prevalence of chronic pain has reached epidemic levels. In addition to personal suffering chronic pain is associated with psychiatric and medical co-morbidities, notably substance misuse, and a huge a societal cost amounting to hundreds of billions of dollars annually in medical cost, lost wages, and productivity. Chronic pain does not have a cure or quantitative diagnostic or prognostic tools. In this manuscript we provide evidence that this situation is about to change. We first start by summarizing our current understanding of the role of the brain in the pathogenesis of chronic pain. We particularly focus on the concept of learning in the emergence of chronic pain, and the implication of the limbic brain circuitry and dopaminergic signaling, which underly emotional learning and decision making, in this process. Next, we summarize data from our labs and from other groups on the latest brain imaging findings in different chronic pain conditions focusing on results with significant potential for translation into clinical applications. The gaps in the study of chronic pain and brain imaging are highlighted in throughout the overview. Finally, we conclude by discussing the costs and benefits of using brain biomarkers of chronic pain and compare to other potential markers.

## Introduction

Chronic pain affects more than 20% of the US adult population (1) and is more prevalent in women than in men (2). Unfortunately, chronic pain does not have a cure or quantitative diagnostic or prognostic tools. Objective measures of disease and response to treatment are necessary for rational and quantitative medical decision making (3). The advent of functional magnetic resonance brain imaging (fMRI) (4) has given a boost to the efforts of understanding the brain neurophysiology of acute and chronic pain as fMRI, along with other techniques such as electroencephalography (EEG), are being intensely applied to the study of various clinical pain populations. These efforts have opened the door for the development of quantitative brain measures of diagnosis, prognosis, and treatment of the disease (5, 6). Here we provide an overview of recent studies advancing potential biomarkers of chronic pain considering our current understanding of the neural pathogenesis of the condition. We discuss the emerging role of the brain limbic system (7) in the pathophysiology of chronic pain and how its role in affective learning and memory can help us develop biologically plausible brain biomarkers for chronic pain. We also touch on the potential economic benefits of brain biomarkers of chronic pain in the context of the staggering cost that this disease is annually engendering (8).

## The Burden of Chronic Pain

Chronic pain is one of the most common reasons adults seek medical care (9). It is also one of the most common causes of disability (10–12), and is associated with major comorbidities like obesity (13) and mental health problems (14) such as depression (15), alcohol (16, 17) and opioid misuse (18). It is estimated that > 50 million American adults live with chronic pain (1) with a staggering annual cost reaching $500–600 billion dollars (19). Low back-pain (LBP) is one of the most prevalent clinical pain conditions (11), with an annual cost reaching $100 billion dollars (20). Osteoarthritis, the most common form of arthritis, affects more than 32.5 million adults in the United States (US), with total annual arthritis-attributable medical care expenditures and earning losses of > $300 billion dollars (21). Inadequately controlled osteoarthritis pain is the primary reason for total joint replacement (22), and available first line analgesic treatments have no (e.g., paracetamol) to small effects (e.g., NSAIDs) over placebo (23). The problem of chronic pain is expected to worsen in the coming decades because the population is getting older. The number of individuals aged above 60 years old is expected to triple by 2050 (24) and age is a major risk factor for developing chronic pain. It is estimated that 50–70% of people over the age of 65 report at least some persistent pain (25–27), and the prevalence of severe pain is higher in the elderly (28). Older adults suffering from low-back pain, for example, are more disabled than their healthy peers (29–31), are more predisposed to frailty (32, 33), and tend to be undertreated (34–36) because of increased difficulty of diagnosis (37) and increased propensity to side effects from analgesics [e.g., non-steroidal anti-inflammatory drugs causing kidney injury (38, 39) or opioids causing increased falls (40, 41)].

Associated with this “population-level pain crisis” is a crisis of opioid analgesic dependence and opioid analgesic overdose death as 450,000 people died from overdoses involving prescription and illicit opioids between 1999 and 2019 in the US (42). These crises are partly a reflection of major gaps in the understanding of the mechanisms of nociception (43), acute, and chronic pain (44) despite significant recent advances (3, 6). Unfortunately, novel pharmacologic treatments for pain have not emerged for some time (44). Together this data indicates that chronic pain is a huge individual and societal burden necessitating further research into mechanism guided novel diagnostic, prognostic, and therapeutic approaches.

## Diagnostic and Prognostic Tools for Chronic Pain

Chronic pain remains a clinical diagnosis based primarily on subjective reports of pain intensity and pain localization (45). Currently, “there are no biomarkers for pain accepted by the US Food and Drug Administration (FDA) or the European Medicines Agency for use in clinical trials (46).” This is a major hurdle in the care of patients suffering from chronic pain because the absence of objective and quantitative tools to diagnose disease, like glucose for diabetes or blood pressure for hypertension, and to measure disease progression or response to treatment, precludes rationale medical decision making. In 1971, the Framingham study identified systolic hypertension as a determinant of long-term cardiovascular risk (47); since then, the reduction of cardiovascular risk by reducing blood pressure (48) and the calculation of risk scores incorporating other objective measures such as cholesterol levels or body mass index (47) have been a major fixture of successful preventive medicine. Instead, most, if not all, the current approaches to treating chronic pain are based on a “trial and error strategy.” This has led to the sad state of affair summarized in the Burden of Chronic Pain section. Hence, the need for objective and quantitative tools to assist clinicians in medical decision making when treating chronic pain patients and to be used as targets or surrogate endpoints in development of new analgesics cannot be more over-emphasized. In addition to the need for quantification in medicine, a mechanism-based approach is critical for treatments and preventions to be impactful (43).

## Pain Perception and the Brain: How Much Do We Know?

**Figure 1:**
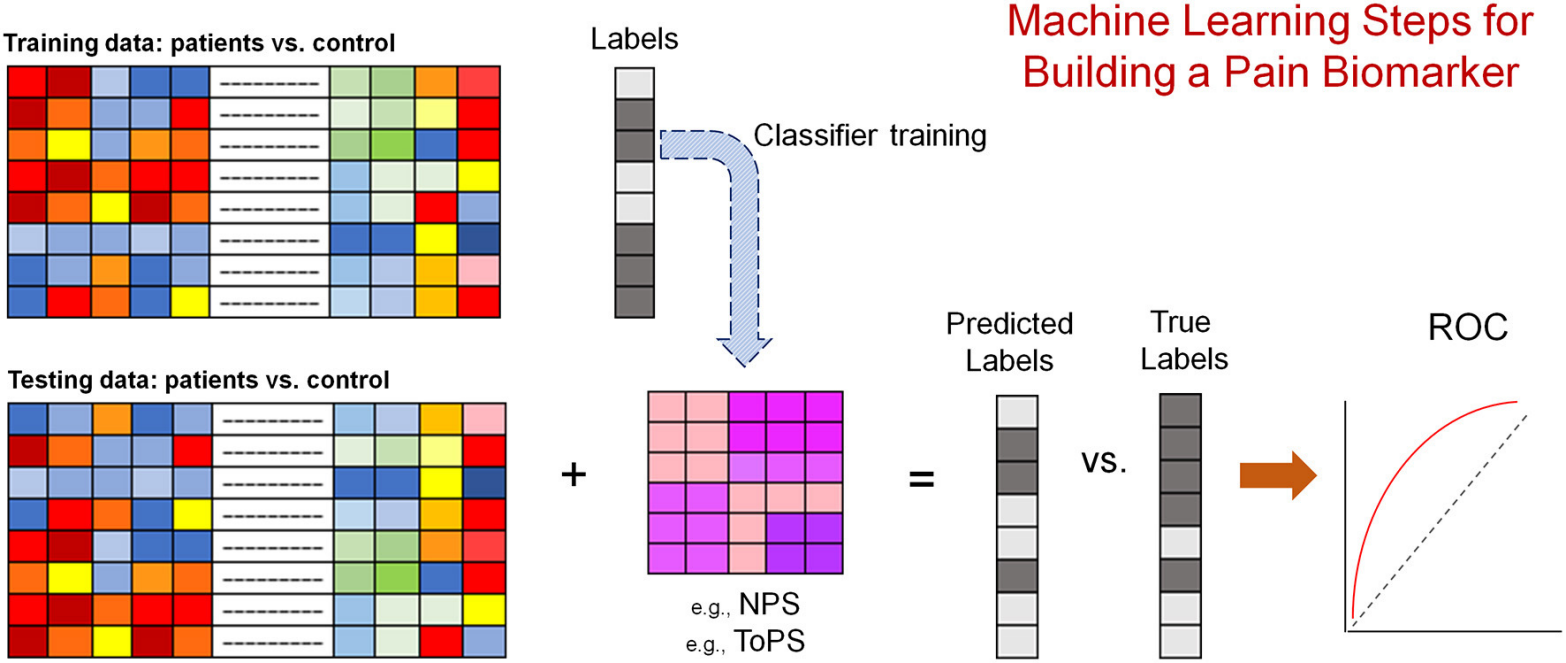
Schematic depiction of the approach used by many papers aimed at developing pain biomarkers. The modality used (i.e., training and testing data) can be the beta maps of the general linear model fit in response to a stimulus [e.g., painful heat (67)], or the connectivity matrix of resting brain activity or structural information [e.g., volume (68, 69)]. Labels (i.e., patient or control) is one example of predictions. Pain intensity is often used as a predicted measure. The most rigorous approach is to keep training and testing data totally separate. However, a lot of the published works out there have used k-fold cross validation approaches.

The lack of understanding of how nociceptive input to the brain gives rise to the conscious perception of pain is a significant knowledge gap in the science of pain (49). It constitutes an obstacle to the discovery of brain biomarkers for chronic pain because the neurophysiology of conscious pain perception is still unknown and consequently the pathophysiology of how this process turns chronic becomes harder to decipher. Unlike touch or vision, which arise because of activity in specific brain tissues (50), pain has very scarce and hard to detect specialized neurons. In addition, the activation of nociceptive input to the brain is not always sufficient or necessary to elicit painful perceptions. This is supported by phenomena such as offset analgesia (51) and the thermal grill illusion (52) suggesting that pain may arise as a result of a pattern of non-nociceptive afferent activations rather than labeled lines of nociceptors (53). Early attempts at identifying a “pain specific” brain tissue in the primary and secondary somatosensory areas (SI and SII) or insula seemed futile (54). Although a more recent cortical stimulation study in humans identified neurons selectively eliciting pain in the posterior insula/SII and adjacent parietal operculum pain responses were very scarce occurring only in 1.4% of all stimulations. The advent of brain imaging (structural and functional magnetic resonance imaging) in the past three decades saw a flurry of studies examining the brain activity associated with acute and chronic pain (55–57). All the same, the physiology of how pain perception arises from nociceptive input is still poorly understood (58). An ensemble of a relatively large number of brain areas are frequently seen to significantly activate in response to acute pain when activity is measured using fMRI (55, 59, 60). An activation likelihood estimation based meta-analysis of acute noxious stimulation fMRI studies showed clusters of activity in the thalamus, basal ganglia, SI, SII, insula/inferior parietal lobule, anterior cingulate cortex (ACC), superior temporal gyrus, and middle and superior frontal gyri, and cerebellum (60). A sub-group of these brain areas (i.e., thalamus, SI, SII, insula and ACC) were dubbed as the “pain matrix” (59) as they are seen in more than 80% of studies of acute pain in healthy subjects (55). However, there is no clear evidence to date that any of these activations are specific to pain perception because the same brain areas observed during painful stimulations are as active during the perception of other salient stimuli in the environment like touch or visual stimuli (61–63), or during negative affective experiences (63), or during salient sensory stimulation in individuals with congenital insensitivity to pain (64). Efforts using novel methods not relying on the general linear model (65) but on the interaction of various brain areas (e.g., functional connectivity) (66) and machine learning (67) are underway to identify specific neural signatures of pain perception (Figure 1). However, the interpretability of such approaches remains limited and has not, to date, significantly advanced our understanding of the physiology of pain perception. Together, the knowledge we accumulated to date about nociceptive processing and pain perception in the brain still cannot explain the neurophysiology of how the former leads to the latter. This unknown is not specific to the perception of pain as it is also unclear how perception of other complex stimuli carrying an incentive salience comparable to pain such as food give rise to feelings of pleasure (i.e., food liking) (70) or flavor constructs (71). This unknown did not however prevent the discovery of reproducible patterns of brain activity and structure changes in chronic pain patients which, although far from explaining the complete picture of the neurophysiology of pain, are nevertheless able to track clinical pain and/or response to treatment. These findings will be discussed below.

## Beyond Sensory Perception: Chronic Pain and Aversive Learning a Conceptualization in Brief

Pain is a sensory and affective experience (72, 73) and engages the limbic brain (7)-in addition to, and part of the “pain matrix or connectome” discussed in the previous section-composed of but not limited to the amygdala, hippocampus, striatum, anterior insula, and prefrontal cortex (60, 63, 67). The limbic system overlaps with the learning circuitry in the brain which integrates motivations and memories to guide behavior (50). As described by Melzack and Casey pain “…becomes overwhelming, demands immediate attention, and disrupts ongoing behavior and thought” (72). To complete the picture this “overwhelming” experience leads to new learning and memory formation with the most likely explanation being to avoid such experiences in the future (74). That nociceptive signals from the periphery eliciting pain lead to learning in animals (75, 76) offers an adaptive advantage is obvious, because animals need to navigate the environment seeking food while at the same time avoiding harm from being attacked or from physical injury (e.g., fall). While the persistence of pain beyond the time needed for healing might be construed as maladaptive recent evidence point to the protective effect of central sensitization in avoiding predation (77). Besides, persistence of pain is thought to be the inevitable consequence of the protective effects of pain as the evolutionary cost of having a hypersensitive nociceptive system protecting animals from injury outweighs the cost of living in chronic pain (78). In both scenarios, the persistence of pain entails ongoing learning as the continuous barrage of afferent nociceptive input entrains the limbic brain and updates memories and associations. Pre-clinical evidence shows in fact that disrupting learning by blocking hippocampal neurogenesis prevents the development of pain behavior in rodent models of inflammatory and neuropathic chronic pain (79). In addition, there is good evidence that persistent pain is associated with new learning (80–82) and altered memories (83) and decision making (84–87). Therefore, the physiological properties of the limbic brain and its plastic response to ongoing pain, which can be measured with multi-modal brain imaging, will directly contribute to the risk of developing chronic pain and the experience of chronic pain, respectively. It stands to reason therefore that diagnostic and prognostic biomarkers of chronic pain should directly involve that circuitry (88, 89). This presents a specificity challenge to the identification of brain biomarkers for chronic pain because the brain circuitry underlying emotions (i.e., limbic) and learning mediates also several other normal and pathological behaviors and traits such as normal memory formation (90) and addiction related learning (91), among others.

The increased likelihood of observing limbic brain areas tracking spontaneous pain (without any outside stimulation) intensity in clinical populations compared to acute pain (elicited by a noxious stimulus) is in fact a distinguishing feature of brain activity collected when chronic pain patients report their spontaneous pain online in the scanner (49, 92–94). Significant evidence exists now showing that activity and functional connectivity in the nucleus accumbens (NAc) and medial prefrontal cortex (mPFC) track chronic low-back pain (CLBP) intensity (95–100). NAc activity tracks also the change in neuropathic pain of patients with post-herpetic neuralgia after treatment (101), is correlated to the number of peripatellar tender sites in knee arthritis patients (102), and shows increased activation during migraine attacks (103). Consistent with fMRI findings, a decrease in μ-opioid binding potential in the NAc have been demonstrated by positron emission tomography (PET) studies across several chronic pain conditions (104–107), indicating either increased binding or decreased baseline levels of internal opioids.

The ventral striatum (including the NAc)-mPFC is the brain network that encodes value of nearly all reward types on a common scale (108). In addition, the accumbens' function is best described as a limbic-motor interface translating motivations into actions (109). Accordingly, healthy pain whether external (e.g., a hot stove) or internal (e.g., belly ache) is a major stimulus requiring the engagement of valuational and motivational circuitry to decide the next step (e.g., moving away from the stimulus or seeking help) (110). Considering this understanding of the role of NAc-mPFC in valuation and motivation under normal conditions the plasticity observed in chronic pain patients emphasizes the valuational and motivational disruptions as key phenotypic expressions of chronic pain consistent with the described clinical picture (111). How this confluence affects the validity of NAc-mPFC based biomarkers in chronic pain has not been explicitly studied and must be investigated in the future. However, existing evidence strongly suggests for example that the valuation signal of acute pain experienced by chronic pain patients is largely different and opposite from the valuation signal of the same stimulus experienced by healthy subjects (96, 112). As such, the NAc activity drops in patients with chronic pain relative to controls during an acute thermal heat pain offset with a large effect size (Cohen's*-d* >>1) (96) suggesting that signals during acute pain might be readily distinguishable from signals experienced in the context of clinical pain.

In addition to NAc and mPFC, amygdala and hippocampus-both major nodes of the limbic brain (7)-have been directly implicated in chronic pain conditions. Hence, amygdala functional connectivity is consistently altered in migraine patients (113–115). Amygdala volume and shape on the other hand are altered in CLBP patients (116, 117). Hippocampal morphology is also changed in chronic pain; CLBP and complex regional pain syndrome patients exhibit decreased hippocampal volume (81) although this finding is not consistent between studies (100). Interestingly, Berger et al. (83) reported that hippocampal morphology predicted pain memory bias in CLBP patients as 77% of the patients exaggerated remembered daily pain. Nociceptive information reaches the limbic brain areas via the spinothalamic-cortical (118), spino-parabrachial-thalmic-cortical (119), spino-parabrachial-amygdala (120) nociceptive projections. A parsimonious model of nociceptive processing would therefore posit that nociceptive information reaching hippocampus and amygdala mediate memory formation and feeds to the NAc and mPFC (74, 76, 121) to guide value based decision making and motor behavior (108, 122). In addition, both the amygdala and the mPFC project to brainstem centers (110) like the periaqueductal gray (PAG) and modulate descending pathways regulating noxious input at the dorsal horn of the spinal cord (110). Given the known anatomy to date, chronic pain can therefore arise either as a result of persistent input from the periphery secondary to injury or inflammation (123) or as a result of gain in the system as the limbic circuitry amplify afferent signals or from the interplay between these two factors (49).

## Brain Biomarkers of Chronic Pain

The FDA-NIH Biomarker Working Group (124) defines a biomarker as “a defined characteristic that is measured as an indicator of normal biological processes, pathogenic processes, or biological responses to an exposure or intervention, including therapeutic interventions. Molecular, histologic, or physiologic characteristics are types of biomarkers. A biomarker is not an assessment of how individual feels, functions, or survives.” The definition implies that, in the case of chronic pain, measures of brain structure or brain activity would be potential biomarkers of disease whereas subjective reports of ongoing pain or elicited pain cannot be biomarkers. This definition does however introduce a logical conundrum into the search for biomarkers for chronic pain and other chronic conditions where subjective report is still the gold-standard like major depressive or anxiety disorders: how can biomarkers be objective if they are derived based on the subjective report of patients to start with? One solution is to rely on big data. Assuming there are objective biomarkers to predict pain pathology and the subjective reported pain intensity is centered around the corresponding pathology level with noise. The problem of identifying important variables with noisily observed responses has been well-studied in statistics, and many famous methods have been developed (125), including LASSO (126) and Elastic Net (127). Theoretical studies have shown that under some regularity conditions, the selected features converge to the true feature as the sample of size goes to infinity (128, 129). With recent advancement in deep learning and convolutional neural networks, automatic feature/biomarker learning becomes possible. However, these methods require large amount of data. For example, in a recently published article, we have shown that a complex model such as a neural network can have much better performance if trained with more brain data (130). Another approach to solving the logical conundrum of biomarker discovery is to test biomarkers in animal models of chronic pain. The same brain activity or structure measure can be obtained in animals confirmed to have an injury (e.g., the spared nerve injury model) and the biomarker then validated in classifying individuals with disease or measuring the extent of pain behavior. Mansour et al. (131) have demonstrated for example that a global measure of disruption of functional connectivity (132, 133) measure correlated to reports of clinical pain intensity in three different types of chronic pain patients (chronic low-back pain, complex regional pain syndrome and knee osteoarthritis) and was also reproducible in a spared nerve injury model of chronic pain in rodents as the disrupted connectivity measure correlated to measures of mechanical allodynia. This approach has the limitation that no subjective reports of pain can be obtained from animals.

Several non-invasive modalities measuring brain structure and activity have been used in the past three decades in pain research (55) and could potentially be used to discover and validate brain-based biomarkers of chronic pain. These modalities include structural and functional magnetic resonance imaging (fMRI), electroencephalography (EEG), magnetoencephalography (MEG), and more recently functional near infrared spectroscopy (fNIRS). While EEG and MEG offer the best temporal resolution, their spatial resolution is limited compared to fMRI. In addition, the location accuracy of EEG, MEG, and fNIRS techniques deteriorates with increasing distance from the scalp, and hence activity in deeper brain structures such as thalamus, striatum or insula, which are important in pain perception (55, 59, 93), might be hard to measure. In contrast, EEG and fNIRS data collection devices are mobile and relatively cheap potentially offering clinicians easy and affordable tools to use.

### Diagnostic Brain Biomarkers

A diagnostic biomarker is “used to detect or confirm presence of a disease or condition of interest or to identify individuals with a subtype of the disease” (124). A review (134) of EEG patterns in patients with chronic pain reported increased theta and alpha power compared to controls but the results are very diverse, and no other EEG studies have validated these findings yet. Interestingly, one of the earliest EEG studies (135) reported *increased* theta power with active treatment but not with placebo and an inverse correlation between change in theta power and change in clinical pain intensity suggesting that the diagnostic EEG patterns of chronic pain patients might be intrinsic and not correctable with analgesia. More recent EEG and MEG studies used machine learning approaches (Figure 1) (136) to discriminate between chronic pain patients and healthy controls (137–140). Some of these studies propose thalamo-cortical dysrhythmia (141) as an underlying pathophysiology of various chronic pain conditions although this dysrhythmia is not specific to chronic pain but is also observed in several other chronic neurologic conditions like chronic tinnitus and depression (137).

Early and consistent findings of altered brain connectivity measured with blood oxygen level dependent (BOLD) (4) fMRI in chronic pain patients (142, 143) suggested a large-scale functional reorganization with biomarker(s) potential. Altered insula to default mode connectivity in chronic pain patients is a notable reproducible finding across studies (96, 97, 144–147), although generalizability of this finding has not been formally tested within one study using separate training and testing data sets. As multivariate data analysis approaches gained traction into the field of brain imaging (148), several potential diagnostic biomarkers were advanced for chronic pain where brain derived classifiers are used to discriminate patients from controls (149) (Figure 1). Both Ung et al. (68), and Labus et al. (69), used multivariate data analysis and validation on held-out samples to identify morphological signatures of chronic pain; their approaches achieved a classification accuracy of 70–76%. Ung et al. (68), used gray matter density derived with voxel morphometry (150) in patients with CLBP, and Labus et al. (69) used cortical thickness derived with FreeSurfer (151) in patients with irritable bowel syndrome, as features to build their predictive models. Mano et al. (152), used brain connectivity (adjacency matrices) and support vector machine to discriminate CLBP patients from healthy controls studied at different sites and achieved an accuracy of 68%. The added contribution of this study was the testing of the predictive model on a previously unseen independent data set rather than on held-out samples only. The authors characterized further brain network changes in CLBP and observed significant modular (153) reorganization of bilateral somatosensory motor cortices in the patients' groups, although how the reorganization of these brain areas contributed to their predictive models was not clear. Lopez-Sola et al. (154) used brain response to painful and non-painful stimuli and the neurologic pain signature (67) weighted pattern of activity to derive a classifier for fibromyalgia patients whose diagnosis relies on testing for hyperalgesia. Authors reported a high sensitivity and specificity (>90%) in discriminating between patients and healthy controls using an out-of-sample validation approach. However, the absence of control chronic pain populations precluded the generalization of these findings to other conditions. An observation common to these studies is the identification of highly distributive predictive brain patterns involving all four lobes of the brain and the cerebellum. In keeping with the thalamo-cortical dysrhythmia theory of chronic pain (155), Tu et al. (156) discovered that CLBP patients dwell longer in a state of increased connectivity between the sub-cortical (including the thalamus) and somatosensory networks and validated their finding using an independent data set. Notably, the dwell time in that hyperconnected state was correlated to pain severity (156).

More recently, Lee et al. (157) built a brain connectivity-based predictive model of tonic pain intensity from fMRI data collected in healthy subjects receiving capsaicin on their tongue, which they dubbed the tonic pain signature (ToPS). Like other multivariate predictive patterns, ToPS was highly distributed across all brain subnetworks with the strongest connections being those between sub-networks, particularly the connections between the somato-motor and fronto-parietal networks. After extensive and rigorous model validations in healthy subjects authors used ToPS to predict clinical pain intensity of sub-acute and chronic low-back pain patients using fMRI data collected at rest and data collected when patients were continuously rating their low-back pain intensity (i.e., task-based data) in the scanner (152, 158). The ToPS predictive performance on the clinical data was mixed. While ToPS' brain response was highly correlated to ratings of SBP intensity using data collected during pain intensity ratings, the correlation was flat when ToPS was obtained from data collected at rest. In contrast, ToPS did not significantly predict ratings of CLBP intensity obtained from data collected during intensity ratings but significantly predicted CLBP intensity when using imaging data obtained at rest. Notably, ToPS response discriminated between patients and healthy controls in two additional and separate brain imaging data sets with an AUC of 73 and 71%, respectively. Lee et al., reported also that ToPS performed better (correlation coefficient-r = 0.48) at predicting low-back pain intensity than models trained on the SBP clinical datasets (*r* = 0.36), but the difference was not statistically significant and the sample size for the clinical validation data sets was relatively smaller (*n* = 35) than the healthy control data sets used to validate ToPS. Besides, ToPS performed better in predicting CLBP pain intensity than the model trained on 17 CLBP patients using the same approach to generate ToPS. As the authors note, ToPS will need to be tested across laboratories and clinical data collected from different pain conditions for further validation before being considered for translational applications.

**Figure 2:**
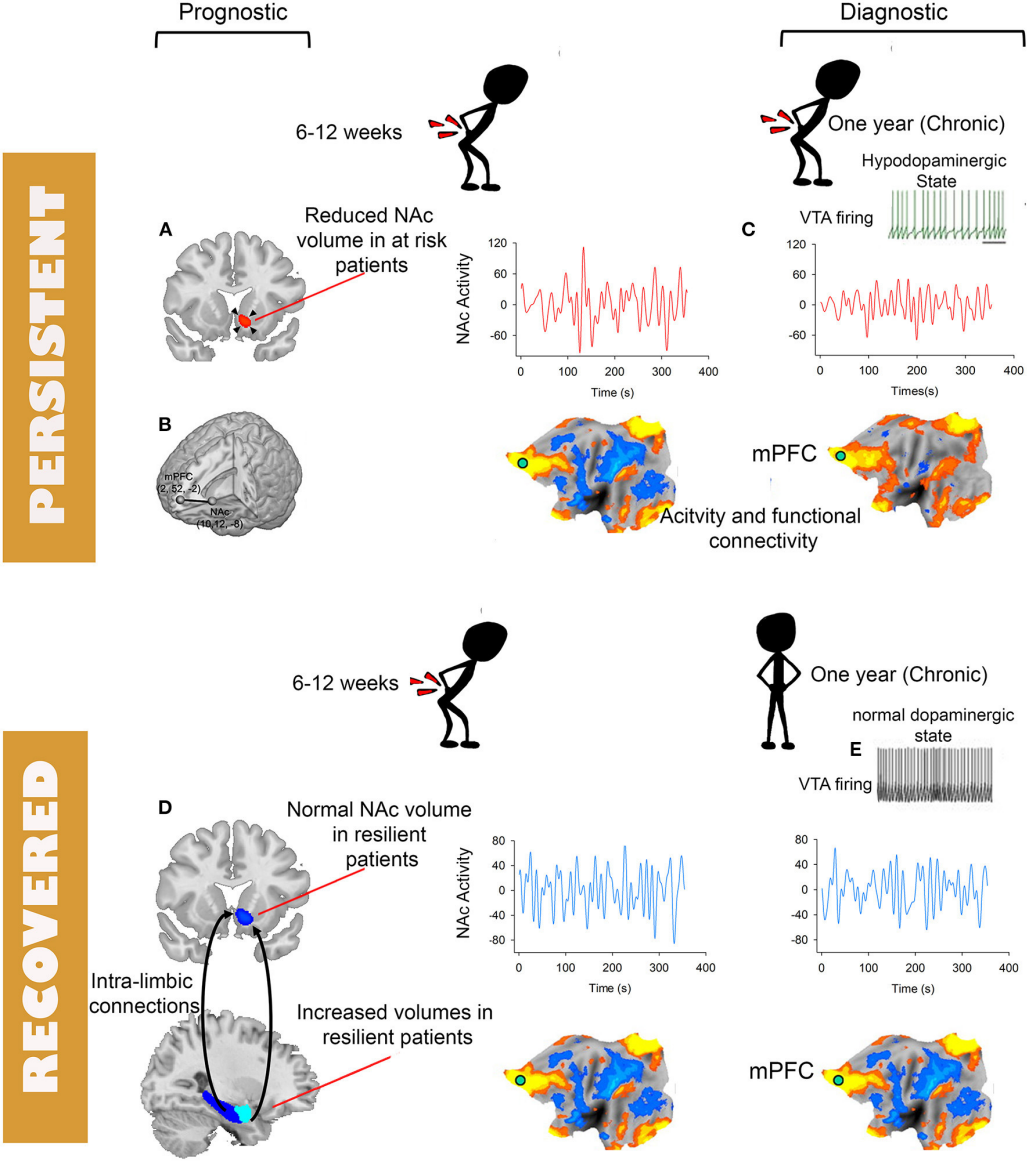
Schematic depiction of brain tissues and modality with potentials for becoming biomarkers for diagnosis and prognosis of chronic pain. Prognostic biomarkers **(A,B,D)** and diagnostic biomarkers **(C)**. The flattened brains are symbolic of various functional plasticity observed in chronic pain including changes in activity, connectivity or multi-variate patterns. Except for the NAc to mPFC functional connectivity **(B)** available prognostic biomarkers are derived from structural MRI **(A,D)**. **(C)** Depiction of loss of low-frequency fluctuations of NAc activity. **(E)** Decreased firing of the ventral tegmental area and the associated hypo-dopamnergic state observed in preclinical and clinical studies of chronic pain.

Cross-sectional studies cannot differentiate causal from consequential brain patterns predictive of chronic pain; therefore, observed diagnostic patterns of chronic pain obtained using cross-sectional approaches are a mixture of both predisposing neural features to chronic pain and plastic changes resulting from living in chronic pain. This highlights the importance of longitudinal studies of the transition from acute to chronic pain where the distinction between causal and consequential brain patterns predictive of chronic pain becomes possible. We have recently identified a neural signature for CLBP that has the potential to become a diagnostic biomarker for this condition (100). Using a combination of a longitudinal design where SBP patients were scanned before and after pain “chronification” or remission and cross sectional cohorts of CLBP studied at different sites, we observed loss of amplitude in the slow-5 (0.01–0.027 Hz) (159) frequency band of NAc activity in CLBP patients. Importantly, the loss of slow-5 was not observed at baseline in SBP patients even if patients are stratified by long-term risk but developed only in SBPp patients after ~1 year of persistent pain. In addition, the loss of slow-5 amplitude was validated in a separate data set pooled from two different sites and discriminated between CLBP patients and healthy controls (AUC > 0.72) from yet another 2 studies **(Figure 2**). Hence, this change in frequency content of NAc activity was absent during the early phase of sub-acute pain, developed as pain became chronic and was highly reproducible across datasets collected at different sites. As we discussed previously the NAc is a hot spot in the pathophysiology of chronic pain as several previous studies pointed to its role in tracking the intensity of pain (95, 96, 98, 101, 158, 160). In addition, pre-clinical data provides neurophysiologic evidence corroborating the role of NAc in chronic pain. Using optogenetic activations, Lee et al. showed that prelimbic (equivalent to mPFC in humans) projections to the NAc in rodents can gate incoming afferent nociceptive input in rodents' models of chronic pain (161). When studied in rodents, acute to chronic pain transition is characterized by decreased dopaminergic signaling between the ventral tegmental area and the NAc, and plastic changes in the cellular structure of medium spiny neurons of the NAc shell (162). Available PET studies of chronic pain patients examining dopamine signaling also suggest the association of chronic pain with a hypodopaminergic state (163–165). The critical involvement of the brain valuation system (108, 166, 167) (e.g., NAc, mPFC) in the plasticity associated with chronic pain is consistent with the often observed disruption of cognitive processes mediated by these brain circuities in chronic pain populations. Chronic pain patients exhibit for example anhedonia (168, 169), disrupted satiety signals (168), and impaired emotional decision making (84, 85, 87). These behavioral impairments indicate in turn that the reproducible changes in the valuation circuitry of patients are biologically explainable and plausible (170) biomarkers of chronic pain.

### Prognostic Brain Biomarkers

A prognostic biomarker is “used to identify likelihood of a clinical event, disease recurrence or progression in patients who have the disease or medical condition of interest” (124).

To date only a handful of studies used fMRI to identify prognostic biomarkers for the transition from acute to chronic pain. Baliki et al. (158) observed that the volume of the NAc measured using voxel-based morphometry shrank in size only in sub-acute low-back pain patients (SBP) (duration 6–12 weeks) who transitioned to chronic pain (SBPp) compared to those who did not (SBPr) and to healthy controls. They also showed that the magnitude of NAc-mPFC functional connectivity is increased in SBPp patients compared to SBPr patients both at baseline and at 1 year follow-up. In an independent (i.e., never “seen” before”) cohort they validated their finding with an area under the curve (AUC) equal to 0.81 when using NAc-mPFC to classify SBPp vs. SBPr at follow-up. Using an expanded sample from the same study they later demonstrated that the morphological properties of the limbic brain predicted the risk of transition from sub-acute to chronic pain (117). They observed that limbic brain white matter connections centered on the dorso-medial and ventro-medial prefrontal cortex, amygdala and hippocampus as well as a smaller volume of the latter two structures constituted independent risk factors for the transition from sub-acute to chronic low-back pain. Combining candidate risk gene single nucleotide polymorphism with the functional and structural properties of the limbic brain in a path analysis allowed them to predict 60% of the variance of the outcome after a sub-acute bout of low-back pain (117). Consistent with these findings we have recently reported using a similar longitudinal design, studying SBP patients at baseline and after transition into chronic pain, that a significantly smaller volume of the NAc in at risk SBPp patients compared to healthy controls predates CLBP; in addition, we corroborated Vachon et al. findings that the volume of amygdala at baseline predicts the risk of transition to CLBP. The volume of NAc was *smaller* in SBPp patients than in healthy controls both at baseline and at follow-up and *smaller* in a cross-sectional cohort of CLBP patients. In contrast, the volume of the amygdala was *larger* at baseline in SBPr patients than in both SBPp patients and healthy controls (Figure 2). This observation suggests that while NAc morphology is a biomarker of risk of pain “chronification,” the morphology of the amygdala is a biomarker of resilience to persisting pain because it was not different from the healthy controls in the at-risk group (i.e., SBPp patients). Mansour et al. reported another potential structural biomarker for resilience to low-back pain chronification using diffusion weighed imaging (DWI) (171). Using fractional anisotropy (FA) measures of white matter diffusion they found that FA values in the superior longitudinal fasciculus and the internal capsule were increased in resilient SBPr patients compared to SBPp patients and healthy controls at baseline when pain was still sub-acute (6–12 weeks duration) and discriminated between SBPp and SBPr patients in an independent cohort an AUC = 0.81. DWI based biomarkers carry a strong potential for translation into clinical applications because they can be easily obtained on hospital scanners in a relatively short period of time (15 min), and have good to excellent test-retest reliability for both intra- and inter-sites repeated measures (172, 173). High reliability is an important and desirable characteristic of biomarkers as it helps in their widespread deployment and generalizability (174). Notably, functional imaging measures that are often considered for biomarkers (131, 157, 158) have lower reliability than DWI measures especially across sites (175–177). A common clinical scenario where DWI of the brain can be added to the work-up would be in patients with low-back pain preparing for spine surgery. The DWI data would then serve to predict the probability of remission or persistence of pain after spine surgery for example where up to 40% of patients report persistent pain post-operatively (178). The DWI scan can be added to the spinal imaging work-up obtained in preparation for surgery and could serve as a quantitative risk assessment to help clinicians and patients make an informed decision about the procedure's outcome. This of course will depend on completing large, and preferably multi-center, clinical trials where DWI brain measures are used to build and validate predictive models of prognosis (e.g., back-pain intensity, or disability) after spine surgery (179).

## Methods of Detection and Clinical Context of Use

The candidate biomarkers we discussed are measured using magnetic resonance imaging (MRI). The approach leverages the ability of MRI to detect a wide range of brain tissue (white and gray matter) and neuronal activity signals in a relatively short data acquisition time and the widespread availability of MRI scanners in medical centers. Blood oxygen level dependent (BOLD) signal is used to measure brain activity and connectivity (4, 180), T1w/T2w weighted images are used to measure subcortical volumes and shapes, and cortical thickness (151, 181), and diffusion weighted imaging (DWI) to measure white matter structure and connectivity (182, 183). These measures can be obtained in a relatively short time 30–60 min, can now be analyzed either online or outsourced easily to biomarker analysis companies in partnership with NIH (184), are non-invasive and low-risk, and require no contrast injection.

In addition to the clinical and economic need that brain biomarkers for chronic pain address in our society they also would be a tremendous help in alleviating discrimination and care inequities in non-communicative patients like new born babies, in patients with communication disabilities, in groups suffering from social bias (185, 186) and in patients where currently available diagnostic tests cannot identify any pathological abnormalities (187) [e.g., a majority of chronic low-back pain patients (188) and patients diagnosed with fibromyalgia (189, 190)]. Rigorously validated brain imaging biomarkers would therefore improve access to treatment and social resources in patients' groups that have been suffering from marginalization in pain treatment (191). Several recent reviews in major pain and neuroscience journals supports the pursuit of brain biomarkers of chronic pain (58, 174, 192–195). Nevertheless, the use of brain biomarkers in an actual clinical scenarios of pain management remain scarce. Harris et al. (196), showed that treatment with pregabalin but not placebo altered insula chemistry and connectivity in patients with fibromyalgia but neither treatment was accompanied by a significant change in clinical pain rating. Most recently, Ashar et al. used fMRI as “an objective correlate of treatment effects” in a clinical trial (NCT0394148) testing pain reprocessing therapy vs. placebo in patients with CLBP (197) and reported decreased anterior middle cingulate cortex activity in response to evoked clinical pain and increased anterior insula to somatosensory cortex connectivity with pain reprocessing therapy more than with placebo treatment. In addition, Reckzeigel et al. (198) recently used brain biomarkers to assess the risk of transition from sub-acute to chronic pain in sub-acute low-back pain patients entering a pharmacological clinical trial (NCT01951105) aimed at preventing the transition to CLBP. The brain based pre-trial risk assessment served to enrich their sample with patients whose risk of recovery was <60%. FMRI was also used to examine objective correlates of treatment effects where authors observed a treatment by sex interaction on the magnitude of NAc-mPFC (198). These are pioneering studies in the field and set the stage for steering the approach to measures in pain treatment clinical trial in a very promising and exciting new direction.

**Figure 3:**
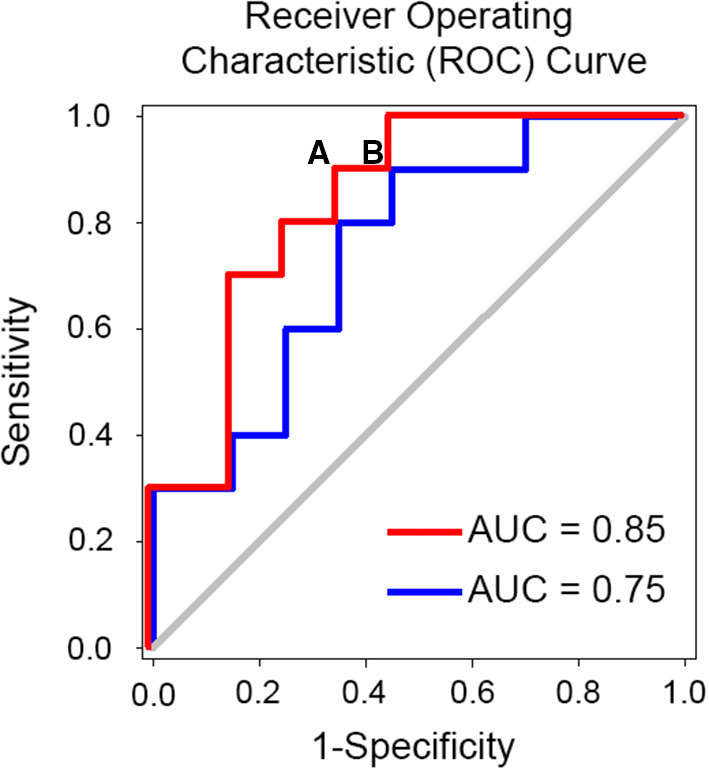
Example of simulated receiver operating characteristic curve (ROC) and identification of optimal cut-off point for decision. An optimal cut off for predicting the success of spine surgery would minimize the false negative rate (i.e., patients who would benefit from the surgery but end up not having it) and minimize the false positive rate (FPR) (i.e., patients who would not have the surgery and end up having it). Note that the false positive rate is equal to 1-specificity and is depicted on the x-axis in the figure. If we consider point **(A)** on the red curve, we will have a biomarker with 90% sensitivity and 34% FPR or 66% specificity. Given that 60% of patients undergoing spine surgery are expected to benefit from it our theoretical biomarker will miss 10% of these patients and hence 6 patients for each 100 patients. Also, given an expected 40% failure rate we expect that ~26 patients will not undergo the surgery anymore. In total, 32 patients who would otherwise undergo surgery without a biomarker-based work-up end up triaged to the no surgery options. If instead we consider point **(B)**, we will have a biomarker with 90% sensitivity and 45% FPR. Following the same calculation 28 patients who would otherwise undergo surgery without a biomarker-based work-up end up triaged to the no surgery options. Abbreviation: AUC, area under the curve.

One criticism for using MRI biomarkers is cost, which varies between $500 and $1,000 for 30 min of brain MRI scanning. To date there are no cost benefit analysis studies to offer guidance on the economic benefits of brain-based biomarkers for chronic pain. Such analysis will depend on the clinical context of use and is beyond the scope of this review. We will present, however, an example of the savings that could be achieved should a prognostic brain biomarker for spine surgery success be translated into clinical use. The literature suggests that spine surgery fails to improve CLBP pain or disability 40% of the time (178). The 2017 Medicare reimbursement rate for a lumbar fusion surgery was at $25,261 in 2017 (199). The estimated utilization rate of lumbar fusion per 1,000 beneficiaries per year was at 20.8 (199). Therefore, the total cost of failed spinal fusions would be 0.4 x 20.8 x $25,261 = $210,172 per 1,000 beneficiaries per year. Assuming a brain-based biomarker can predict success of the surgery with a 90% sensitivity and 66% specificity (Figure 3), the number of patients undergoing surgery will be reduced by 32%, which can save 0.32×20.8 × $25,261 - 20.8 × $1,000 = $147,337, per 1,000 beneficiary per year, where 20.8 × $1,000 is MRI related cost. This is an underestimation of the saving because the subsequent medical cost that the patients who fail the surgery will incur throughout their life is not considered. While this benefit comes at the risk of leaving out a small number of patients without surgery, who would have otherwise benefited if they had the surgery because they fall in the false negative range, it is an example of how such tools could be used to help both clinicians and patients gauge the risk of success and failure and help them come to a decision. MRI biomarkers can also provide novel standardized measures of endpoints for phase II and III clinical trials (200–202), which rely mostly on subjective pain ratings, and novel targets for reverse translational animal studies for drug developments (161, 201, 203). For example, a standardized volumetric, shape or activity measure [e.g., hippocampus (117), NAc (203)] would provide a quantitative and reliable tool for clinical pain prognosis/diagnosis. In addition, a diagnostic biomarker of chronic pain can be used as a surrogate endpoint in clinical trials to test if they are changed by analgesia and to help make medical related decision making (e.g., spine surgery). On the other hand, prognostic brain biomarkers can help identify high risk patients for chronic pain in clinical trials and hence decrease sample size requirements by targeting specifically these patients.

## Comparison to Other Pain (BIO-) Markers

Brain MRI based biomarkers offer the advantage of being part of the *specific* pathogenic process leading to chronic pain (46, 58, 174), the nervous tissue including the brain being the biological substrate of chronic pain independently from any subjective psychological reports. Once validated, brain MRI based biomarkers are therefore more amenable to reverse translation to animal research, and hence novel analgesic targets development, than quantitative sensory testing (QST) or psychosocial phenotyping (204), which depend on subjective patient reports, are unobtainable in animals. Furthermore, the direct access of brain imaging techniques to brain structure and physiology of patients' brain tissue relative to other approaches lends it more potential for specificity. For example, when different types of chronic pain patients report their stimulus-free spontaneous pain in the magnet they show different corresponding functional maps (92). Similarly, altered brain networks in chronic pain patients differ between different clinical pain syndromes (99, 114, 144). In contrast, no QST profile is specific to a given clinical pain condition (204). In addition, studies using QST to differentiate pain patients from healthy controls (205, 206) or using QST for prognostic profiling of pain patients (207) are still conflicting. Recent data has also shown that chronic pain and disability can be reduced with no associated change in QST profiles (208), and QST profiles can improve with treatment without a significant change in spontaneous subjective clinical pain (196). However, some evidence suggests that QST can predict response to chronic pain treatments (209). Compared to other biological markers of clinical pain such as genetic profiling, MRI based biomarkers have seen a faster progress (5, 174, 210, 211) and are closer to adoption in clinical trials (145, 191, 198, 212). As such progress in identifying reproducible diagnostic or prognostic genetic polymorphism for chronic pain has been limited so far to rare causes of chronic pain such as gain of function mutations of the sodium channel causing inherited erythromelalgia (213). Although more common chronic pain conditions like chronic low back pain or migraine headaches have a significant heritability (214, 215) a gene-based diagnostic biomarker, for example, is difficult to establish because these conditions are polygenic (216) hence requiring very large sample sizes for replicability, which, to date, remains limited (5, 210). The availability of large data banks with genetic information such as the UKBioBank (https://www.ukbiobank.ac.uk/) will hopefully accelerate the development of genetic biomarkers for chronic pain (217). In contrast, brain MRI based biomarkers might be more expensive than QST, psychosocial assessments, or genetic testing, and their analysis in remote medical centers might necessitate outsourcing. Regardless, the development of all these (bio)-markers of chronic pain are not mutually exclusive and will hopefully be combined to better predict outcomes.

## Conclusion

The use of brain imaging to discover biomarker for chronic pain has a reached an exciting period as we inch closer to translate experimental findings into clinical use. The accumulation of neuroimaging repositories will tremendously help with this effort, emphasizing the need for pain scientists to share their data to allow biomarker validations across sites. Other brain imaging approaches targeting glial physiology in humans (218) and imaging of animal models of chronic pain (131, 219–221) will also help in further developing biomarkers for chronic pain and in invasively studying their sub-components.

## Author Contributions

PG and ZZ drafted the brain imaging sections of the manuscript. JG drafted the sections on non-imaging biomarkers. All authors edited the final version.

## Funding

This work was supported by grants from National Institute on Drug Abuse K08DA037525, National Institute of Neurologic Disorders and Stroke R21NS1188162, Yale University Department of Psychiatry, and University of Rochester Department of Psychiatry.

## Conflict of Interest

The authors declare that the research was conducted in the absence of any commercial or financial relationships that could be construed as a potential conflict of interest.

## Publisher's Note

All claims expressed in this article are solely those of the authors and do not necessarily represent those of their affiliated organizations, or those of the publisher, the editors and the reviewers. Any product that may be evaluated in this article, or claim that may be made by its manufacturer, is not guaranteed or endorsed by the publisher.
